# Macroscopically entangled light fields

**DOI:** 10.1038/s41598-021-90694-6

**Published:** 2021-05-31

**Authors:** Byoung S. Ham

**Affiliations:** Center for Photon Information Processing, School of Electrical Engineering and Computer Science, Gwangju Institute of Science and Technology, 123 Chumdangwagi-ro, Buk-gu, Gwangju, 61005 South Korea

**Keywords:** Quantum mechanics, Quantum optics

## Abstract

A novel method of macroscopically entangled light-pair generation is presented for a quantum laser using randomness-based deterministic phase control of coherent light in a coupled Mach–Zehnder interferometer (MZI). Unlike the particle nature-based quantum correlation in conventional quantum mechanics, the wave nature of photons is applied for collective phase control of coherent fields, resulting in a deterministically controllable nonclassical phenomenon. For the proof of principle, the entanglement between output light fields from a coupled MZI is examined using the Hong-Ou-Mandel-type anticorrelation technique, where the anticorrelation is a direct evidence of the nonclassical features in an interferometric scheme. For the generation of random phase bases between two bipartite input coherent fields, a deterministic control of opposite frequency shifts results in phase sensitive anticorrelation, which is a macroscopic quantum feature.

## Introduction

Since the seminal paper by Einstein, Polodsky, and Rosen (EPR) in 1935^1^, the so-called spooky action of nonlocal correlation has been intensively studied for the fundamental understating of quantum mechanics^1–16^. For direct evidence of nonclassical features in entangled photon pairs, the Bell inequality violation^2^, Franson-type nonlocal correlation^3^, and Hong-Ou-Mandel (HOM) anticorrelation^4^ have been investigated over the decades in both noninterferometric^5–9^ and interferometric schemes^10–16^. In these studies, not only entangled photon sources from spontaneous parametric down conversion (SPDC) processes^17^, but also independent light sources from such as quantum dots and sunlight^18^ have been used for demonstrating nonclassical features via coincidence measurements. However, all of these studies have focused on the particle nature of photons, even though coherence is the bedrock for entanglement generation. Providing entangled photon pairs is an essential step toward quantum information processing via controlled-NOT gate operations^19^, entanglement swapping^20^, quantum teleportation^21^, and unconditionally secured key distribution^22^. Multiphoton-based bipartite entanglement of a N00N state^23^ or a Schrodinger’s cat^24^ is essential for quantum sensing applications to beat the standard quantum limit. Unfortunately, however, there is no recipe for entangled photon-pair generation. The generation of macroscopic quantum states with large ${\text{N}} > 100$ may not be technically possible with current technologies^25^.

Recently, the fundamental physics of quantumness or nonclassicality has been investigated for a HOM dip^26^, photonic de Broglie wavelength^27^, and Franson-type nonlocal correlation^28^ using the wave nature of photons, where the origin of anticorrelation in a HOM dip is rooted in a ${\uppi }/2$ phase shift between the entangled photons^26,29^. The origin of nonlocal correlation has been discovered in the basis randomness for a coupled bipartite system via quantum superposition^30^. Unlike the particle nature of photons limited to coincidence detection, however, the wave nature of photons emphasizes coherence. Here, coherence represents a typical interference such as in Young’s double slits. Such coherence has also been demonstrated in an MZI for single photons^31^. Collective phase control of an atomic ensemble has already been demonstrated for quantum interface^32–36^. Likewise, collective phase control of ensemble photons from a laser is a key technique in the present manuscript, resulting in inherent macroscopic quantum manipulation via the orthonormal basis randomness of the coupled system^27–30^. Here, we present a novel theory of macroscopically entangled light-pair generation using the randomness of the phase basis in an MZI. Considering the coherence de Broglie wavelength (CBW)^27^, the origin of macroscopically entangled light pairs is the superposition between MZI phase bases^26–30^, where randomness is an essential requirement for $g^{\left( 1 \right)}$ coherence^37^. According to the basic quantum physics, the second-order intensity correlation $g^{\left( 2 \right)}$ is closely related with the first-order correlation $g^{\left( 1 \right)}$ in coherence optics, where $g^{\left( 2 \right)} = g^{\left( 1 \right)} + 1$^38^. Here, Heisenberg’s uncertainty principle does not limit a quantum mechanically coupled system as it does in EPR^1^ and Popper’s though experiment^39^.

## Results

**Figure 1:**
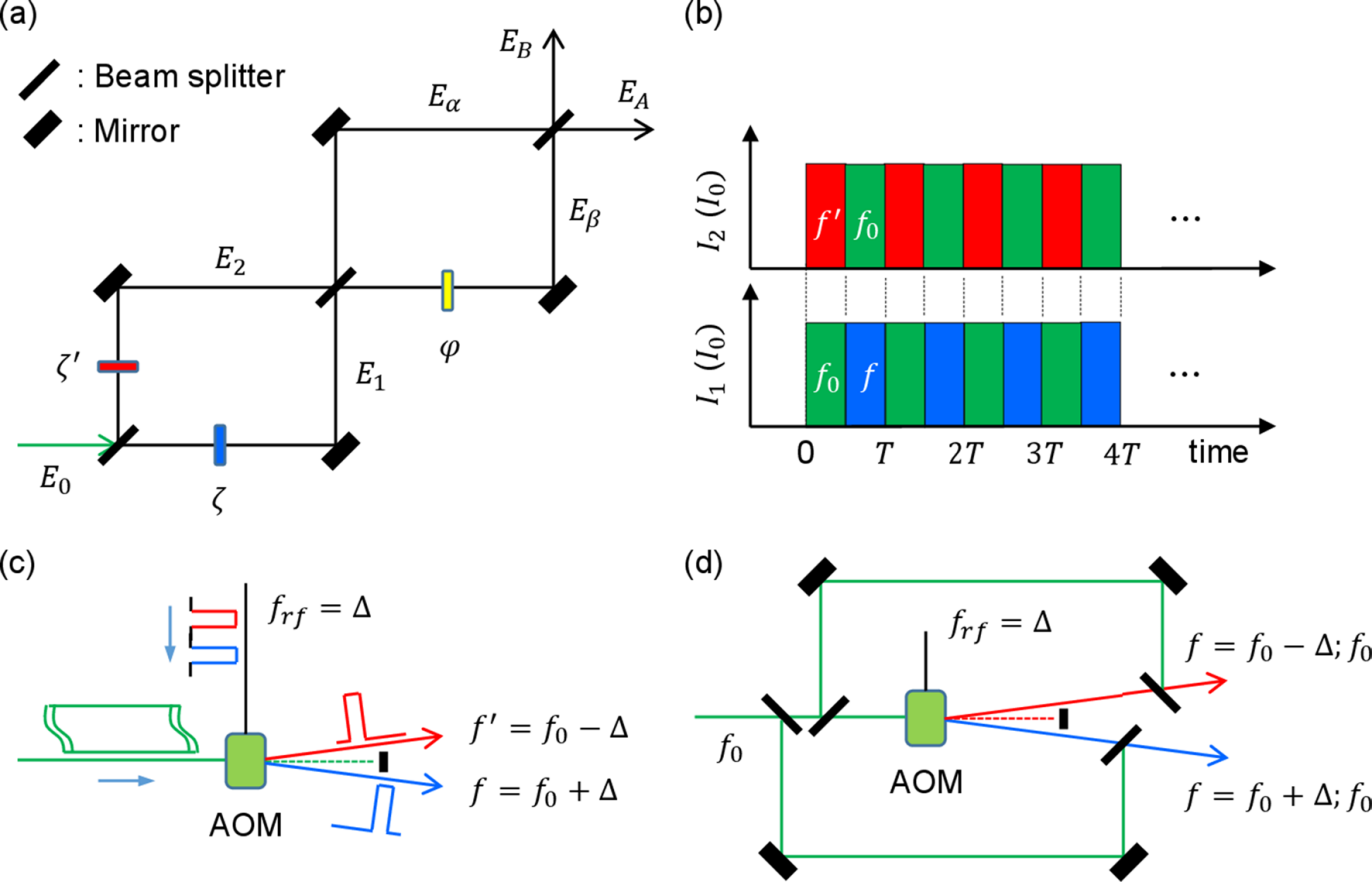
Schematic of macroscopic entangled field generation. (**a**) A Mach–Zehnder interferometer for Hong-Ou-Mandel type proof. (**b**) Alternative pulse sequence. (**c**) Symmetric detuning. (**d**) Superposition for basis randomness.

Figure 1 shows schematics of the macroscopically entangled light-pair generation in an MZI by providing its phase basis randomness. As is already known, basis randomness is an essential requirement of quantum superposition between bipartite systems such as in Young’s double slits and an MZI^37^. Once basis randomness fails, there is no quantum superposition but instead classical superposition^40^. Here, it should be noted that the conventional understanding of classicality for the individuality of coupled photons has been discussed in Bell’s inequality theorem^2^. In that sense, coherence optics may or may not belong to classical physics depending on the phase choice, as discussed for anticorrelation^26,29^.

Figure 1 is for pure coherence optics, where the first MZI in Fig. 1a is a preparation stage for the random phase bases between two input fields $E_{1}$ and $E_{2}$ by classically controlling the symmetric phases ${\upzeta }$ and $\zeta^{\prime}$. The original input field $E_{0}$ in Fig. 1a is for typical laser light, and a single photon case is also included for the present analysis. For the present scope, however, we set $E_{0}$ as a commercially available laser light for the discussion of macroscopic quantum features. Figure 1b is a phase-controlled light pulse sequence for $E_{1}$ and $E_{2}$, where $E_{1}$ and $E_{2}$ are designed to be symmetrically detuned by $\pm {\Delta }$ $\left( {{\text{blue}}\;{\text{and}}\;{\text{red}}} \right)$, respectively, in a frequency domain across the center frequency $f_{0}$ of $E_{0}$ (green). Here, $I_{j}$ represents the corresponding intensity of the field $E_{j}$, where the detuned fields ($E_{1}$ and $E_{2}$) are alternatively coupled with the original field $E_{0}$ (see Fig. 1c,d). For example, if $E_{1} { }\left( {E_{2} } \right)$ is turned on, $E_{2} { }\left( {E_{1} } \right)$ must be turned off and replaced by $E_{0}$. For the input fields $E_{1}$ and $E_{2}$, the symmetric phase pair, ${\upzeta }$ and $\zeta^{\prime}$, is provided by the product of the detuning $\pm \Delta$ and the pulse duration T/2: $\upzeta = \Delta {\text{T}}/2;{\upzeta ^{\prime}} = - \Delta {\text{T}}/2$. Figure 1c shows how to generate symmetric detuning $\pm {\Delta }$ using an acousto-optic modulator (AOM) driven by an rf-field generator. Figure 1d shows how both oppositely diffracted pulses are alternatively selected and combined with the original one, as seen in Fig. 1b. All controls are classical, deterministic, and compatible with current optoelectronic technologies.

### Theory

Based on Fig. 1, we now present a novel theory of macroscopically entangled light-pair generation. Using matrix representations for coherence optics, the following relations are obtained (see Section 1 of the Supplementary Information):

1 $$E_{A} = \frac{{E_{0} }}{2\sqrt 2 }\left[ {e^{i\zeta } \left( {1 - e^{i\varphi } } \right) - e^{{ - i\zeta^{\prime}}} \left( {1 + e^{i\varphi } } \right)} \right],$$

2 $$E_{B} = \frac{{iE_{0} }}{2\sqrt 2 }\left[ {e^{i\zeta } \left( {1 + e^{i\varphi } } \right) - e^{{ - i\zeta^{\prime}}} \left( {1 - e^{i\varphi } } \right)} \right].$$

where $\zeta = {\Delta T}/2$, and $\zeta^{\prime} = - \zeta$. The detuning ${\Delta }$ is with respect to the center frequency $f_{0}$ of the input field $E_{0}$, as shown in Fig. 1c, by an acousto-optic modulator (AOM) driven by an rf frequency at $f_{rf} \left( { = {\Delta }} \right)$, in which $f = f_{0} + {\Delta }$ and $f^{\prime} = f_{0} - {\Delta }$. As a result, the corresponding intensities of the output fields are obtained:

3 $$I_{A} = \frac{{I_{0} }}{2}\left[ {1 - \sin \left( \varphi \right)\sin \left( {\zeta ;\zeta^{\prime}} \right)} \right],$$

4 $$I_{B} = \frac{{I_{0} }}{2}\left[ {1 + \sin \left( \varphi \right)\sin \left( {\zeta ;\zeta^{\prime}} \right)} \right],$$

where $\sin \left( {\zeta ;\zeta^{\prime}} \right)$ stands for a mutually exclusive state, i.e., either $\sin \left( \zeta \right)$ or $\sin \left( {\zeta^{\prime}} \right)$ at a time via superposition with the original field as shown in Fig. 1c,d. The symmetric detuning control of $\pm {\Delta }$ by an AOM is for toggle switching between $f$ and $f^{\prime}$ as shown in Fig. 1b. Thus, each mean value of the output intensity becomes uniform at $\left\langle {I_{A} } \right\rangle = I_{0} /2$ and $\left\langle {I_{B} } \right\rangle = I_{0} /2$ if $\zeta = \left( {2n + 1} \right)\pi /2$ and $\zeta^{\prime} = - \zeta$, i.e., ${\Delta T} = \left( {2n + 1} \right)\pi /2$, where ${\text{T}}/2$ is the pulse duration of $E_{1}$ and $E_{2}$. Once again, the modulated and superposed fields, $E_{1}$ and $E_{2}$, are accompanied by $E_{0}$ for basis randomness, as shown in Fig. 1b,d.

Finally, the intensity product R of the output fields in Fig. 1a is as follows:

5 $${\text{R}} = I_{A} I_{B} = \left[ {1 - \sin^{2} \left( \varphi \right)\sin^{2} \left( {\zeta ;\zeta^{\prime}} \right)} \right].$$

**Figure 2:**
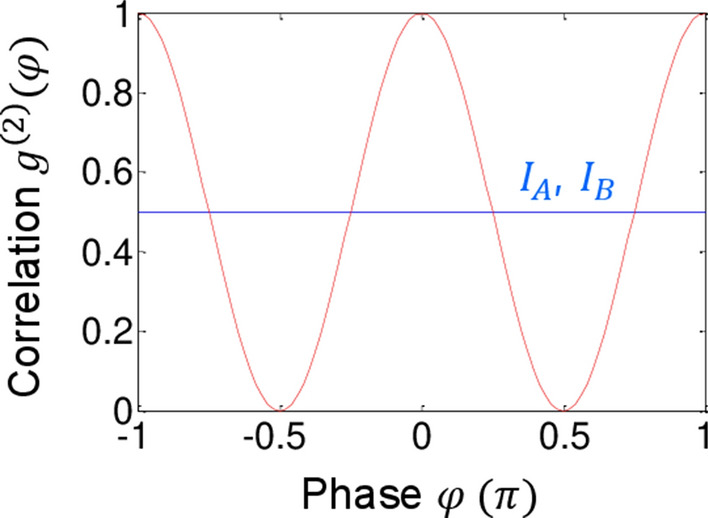
Numerical calculations for intensity correlation $g^{\left( 2 \right)} \left( \varphi \right)$. The phase ${{\upvarphi }}$ is within coincidence detection ${{\upvarphi }} \in \left\{ {{\upzeta },\zeta^{\prime}} \right\}$. The blue line is a classical lower bound.

In Eq. (5), ${\text{R}} = \left[ {1 - \sin^{2} \left( \varphi \right)} \right]$, corresponding to the coincidence detection in the particle nature of photons, is satisfied for the specific condition of symmetric phase control with $\zeta = \left( {2n + 1} \right)\pi /2$ and $\zeta^{\prime} = - \zeta$, where this result is deterministic and a single-shot measurement. Although the mean values of $I_{A}$ and $I_{B}$ are constant at $I_{0} /2$, the product R sinusoidally oscillates as a function of ${\upvarphi }$. This is the quintessence of the present theory for nonclassical features of anticorrelation in a macroscopic regime, resulting in:

6 $$g^{\left( 2 \right)} \left( \varphi \right) = \frac{{\left\langle {I_{A} I_{B} } \right\rangle }}{{\left\langle {I_{A} } \right\rangle \left\langle {I_{B} } \right\rangle }} = \frac{1}{2}\left\langle {1 - \sin^{2} \left( \varphi \right)} \right\rangle ,$$

where conventional variable $\uptau$ for coincidence measurements is now replaced by ${\upvarphi }$ for coherence measurements due to its higher sensitivity of coherent photons. Equation (6) is robust with respect to the laser bandwidth ${{\updelta \upomega }}$ ($c\delta \omega^{ - 1} \gg\lambda )$ and thus shows a definite evidence of coherence-based quantum correlation in an interferometric scheme. The degree of quantum correlation in Fig. 2 is deterministically measured by the control of phase ${\upvarphi }$.

Here, our concern is about the inputs fields of $E_{\alpha }$ and $E_{\beta }$ whether they are entangled or not for Fig. 2. It should be noted that $e^{i\zeta }$ and $e^{{i\zeta^{\prime}}}$ in Fig. 1a are mutually exclusive as shown in Fig. 1b. If both inputs $E_{\alpha }$ and $E_{\beta }$ are represented in a form of $\left| \psi \right\rangle = \left| {E_{\alpha } } \right\rangle \left| {E_{\beta } } \right\rangle$ for a field(photon)-path relation under the mutually exclusive condition, the following equation is obtained (see Section 2 of the Supplementary Information):

7 $$\left| \psi \right\rangle = \frac{{iE_{0} }}{\sqrt 2 }\left( {\left| 1 \right\rangle_{\alpha } \left| 0 \right\rangle_{\beta } - ie^{i\varphi } \left| 1 \right\rangle_{\alpha } \left| 0 \right\rangle_{\beta } } \right).$$

Equation (7) shows Bell states. Thus, the input fields $E_{\alpha }$ and $E_{\beta }$ are macroscopically entangled via random choice of the phase ${\upzeta }$ and ${{\upzeta^{\prime} }}\left( { = - {\upzeta }} \right)$ with a specific value. Because MZI has no discrepancy between a particle and a wave, the inputs of $E_{\alpha }$ and $E_{\beta }$ are satisfied for both a single photon and a coherent laser field. This is the essence of the macroscopic entanglement generation in the present paper.

Figure 2 shows numerical calculations for Eq. (6). As analyzed above, each output field’s mean value is fixed at $I_{0} /2$ by an alternative selection of $\pm \frac{{\uppi }}{2}$ phase-shifted ${\upzeta }$ and $\zeta^{\prime}$ using $\pm {\Delta }$ frequency control. Here, the intensity correlation $g^{\left( 2 \right)} \left( \varphi \right)$ covers both classical ($g^{\left( 2 \right)} \left( \varphi \right) \ge 0.5$) and quantum ($g^{\left( 2 \right)} \left( \varphi \right) < 0.5$) regimes depending on the ${{\upvarphi }}$ values. This is a unique feature of the wave property governed by the field’s wavelength ${\uplambda }$-dependent path-length difference. Considering an actual bandwidth of coherent light $E_{0}$, however, Eq. (6) may result in dephasing-caused partial washout of the $g^{\left( 1 \right)}$ effect in $g^{\left( 2 \right)}$. Unlike the SPDC case with random phases among entangled photon pairs due to the intrinsic frequency detuning swapping^29^, the bandwidth-caused dephasing in Eq. (6) is far less sensitive to dephasing. Thus, the present method of the macroscopically entangled light-pair generation is robust to laser sources. Experimental results will be presented elsewhere.

### Discussion

In Fig. 1a, the specific condition for ${\upzeta } = \pm {\uppi }/2$ and ${{\upzeta^{\prime}}} = - {\upzeta }$ is to compensate the BS-caused phase shift of ${\uppi }/2$^41^, resulting in uniform intensity distribution for $E_{\alpha }$ and $E_{\beta }$ via random field bunching between $E_{1}$ and $E_{2}$:

8 $$E_{1} = \frac{{E_{0} }}{\sqrt 2 }\left[ {1 - {\text{cos}}\left( {\zeta ;\zeta^{\prime}} \right)} \right],$$

9 $$E_{2} = \frac{{E_{0} }}{\sqrt 2 }\left[ {1 + {\text{cos}}\left( {\zeta ;\zeta^{\prime}} \right)} \right].$$

Due to the symmetric cosine function, Eqs. (8) and (9) have no effect on the symmetric phase shift of $\pm {\Delta }$, resulting in $I_{\alpha } = I_{\beta }$ regardless of ${{\upvarphi }}$. This is equivalent to random swapping of symmetric frequency detuning in the SPDC-generated photon pairs^29^. Thus, $E_{\alpha }$ and $E_{\beta }$ impinging on a BS exhibit macroscopic quantum features, similar to a HOM dip with entangled photon pairs as derived in Eq. (7). For the proposed coherence anticorrelation in Fig. 1, the interference between $E_{A}$ and $E_{B}$ is φ- dependent, resulting in anticorrelation at ${{\upvarphi }} = \pm \frac{\pi }{2}\left( {2n + 1} \right)$. Thus, the ${\upzeta }$-controlled MZI in Fig. 1a acts as a quantum device whether the input field is a single photon or coherent light. As already discussed, anticorrelation in a HOM dip naturally satisfies the phase basis relation in a particular system^26,29^. For a BS, the phase bases for anticorrelation are $\pm {\uppi }/2$, while for an MZI, it is 0 and ${\uppi }$. In Fig. 1a, the phase basis is modified due to the ${\upzeta }$ condition from $\pm {\text{n}} \uppi$ to $\pm {\uppi }/2$.

According to Heisenberg’s uncertainty principle or de Broglie’s wave-particle duality^42^, conventional emphasis on the particle nature of photons is a matter of preference depending on the light source. Recently, a dynamic (encounter) delayed-choice method has been demonstrated for the wave property of a photon^43^. Unlike SPDC-generated entangled photon pairs, the coherent light source in Fig. 1 has the benefits of determinacy and controllability. Due to such benefits of coherence optics, the confirmed entangled light pair $E_{\alpha }$ and $E_{\beta }$ can be extracted from the MZI system by inserting a BS into each arm, while keeping the same anticorrelation measurements for $E_{A}$ and $E_{B}$ in Fig. 1a. This on-demand control of quantum correlation based on the coherent field-based intrinsic property of the wave nature of photons is the fundamental difference and novelty of the present paper. Compared with a typical laser system, this entangle light pair is called a quantum laser. Quantum mechanics is not as mysterious anymore in a coupled system, but instead can be definite and imperative as Einstein dreamed.

Regarding potential applications, the proposed method can be applied for a quantum laser whose light pair is macroscopically entangled, satisfying a N00N state with unbounded N. Compared with the MZI-superposition-based coherent de Broglie wavelength^27,30^, the quantum laser has an additional benefit of robustness in phase fluctuations. However, the unbounded N in the quantum laser is post-selective by using the particle nature of photons, otherwise bipartite entangled photon states (N = 2) dominate according to Poisson distribution (discussed elsewhere). The quantum laser may be applied for a quantum Lidar in quantum sensors, quantum keys in a quantum key distribution, and even a photonic qubit in quantum computations. Compared with amplitude-limited modulation in conventional quantum information, the proposed method may open the door to quantum phase modulation as well as quantum wavelength division multiplexing. These applications are unprecedented and macroscopic in nature.

## Conclusion

In conclusion, a novel method for macroscopically entangled light-pair generation was proposed, analyzed, and numerically demonstrated for both fundamental understanding of quantum mechanics and potential applications in future coherence-based quantum technologies. Unlike conventional understanding on quantum mechanics based on the particle nature of photons, the control of a coherent photon ensemble in the present analysis is phase deterministic in an MZI system for macroscopic quantum features. Owing to the wave nature of photons, coherence has also an inherent benefit of collective control, resulting in macroscopic quantum manipulation. The proposed method is compatible with coherence optics. The essential requirement for macroscopic quantum features is quantum superposition based on random phase bases, satisfying indistinguishability in $g^{\left( 1 \right)}$ coherence as well as $g^{\left( 2 \right)}$ correlation. In other words, manipulation of macroscopic indistinguishability is a fundamental bedrock of quantum features that are achievable coherently. As defined in Bell’s inequality, $g^{\left( 1 \right)}$ coherence has to be distinguished from classicality based on individual particles.

## Supplementary Information

Supplementary Information.
