# Supplementary material for: Macroscopically entangled light fields
Source: Sci Rep. 2021 May 31;11:11388. doi: 10.1038/s41598-021-90694-6 (PMC8167127; doi:10.1038/s41598-021-90694-6)
Supplement: Supplementary file 1 — Supplementary Information. [file 41598_2021_90694_MOESM1_ESM.pdf]

Supplementary Information for

Macroscopically entangled light fields

BS Ham

1. Matrix representations for equations (3) and (4) (see Fig. 1)

$$\begin{bmatrix} E_A \\ E_B \end{bmatrix} = [BS][\Phi][BS][Z][BS]\begin{bmatrix} E_0 \\ 0 \end{bmatrix}$$

$$= \frac{1}{2\sqrt{2}}\begin{bmatrix} 1-e^{i\varphi} & i(1+e^{i\varphi}) \\ i(1+e^{i\varphi}) & -(1-e^{i\varphi}) \end{bmatrix}\begin{bmatrix} e^{i\zeta} & ie^{i\zeta} \\ ie^{-i\zeta'} & e^{-i\zeta'} \end{bmatrix}\begin{bmatrix} E_0 \\ 0 \end{bmatrix}$$

$$= \frac{1}{2\sqrt{2}}\begin{bmatrix} e^{i\zeta}(1-e^{i\varphi}) - e^{-i\zeta'}(1+e^{i\varphi}) & i[e^{i\zeta}(1-e^{i\varphi}) + e^{-i\zeta'}(1+e^{i\varphi})] \\ i[e^{i\zeta}(1+e^{i\varphi}) - e^{-i\zeta'}(1-e^{i\varphi})] & -e^{i\zeta}(1+e^{i\varphi}) - e^{-i\zeta'}(1-e^{i\varphi}) \end{bmatrix}\begin{bmatrix} E_0 \\ 0 \end{bmatrix}, \quad \text{(S1)}$$

where $[BS] = \frac{1}{\sqrt{2}}\begin{bmatrix} 1 & i \\ i & 1 \end{bmatrix}$, $[\Phi] = \begin{bmatrix} 1 & 0 \\ 0 & e^{i\varphi} \end{bmatrix}$, $[Z] = \begin{bmatrix} e^{i\zeta} & 0 \\ 0 & e^{-i\zeta'} \end{bmatrix}$, $\zeta = \Delta\tau$, and $\zeta' = -\Delta\tau$.

The corresponding intensities are as follows:

$$I_A = \frac{I_0}{8}[e^{i\zeta}(1-e^{i\varphi}) - e^{-i\zeta'}(1+e^{i\varphi})][e^{-i\zeta}(1-e^{-i\varphi}) - e^{i\zeta'}(1+e^{-i\varphi})],$$

$$= \frac{I_0}{8}[(1-e^{i\varphi})(1-e^{-i\varphi}) + (1+e^{i\varphi})(1+e^{-i\varphi}) - e^{i(\zeta;\zeta')}(1-e^{i\varphi})(1+e^{-i\varphi}) - e^{-i(\zeta;\zeta')}(1+e^{i\varphi})(1-e^{-i\varphi})]$$

$$= \frac{I_0}{8}[2 - 2cos\varphi + 2 + 2cos\varphi - e^{i(\zeta;\zeta')}(-2isin\varphi) - e^{-i(\zeta;\zeta')}(2isin\varphi)]$$

$$= \frac{I_0}{2}[1 - sin(\varphi)sin(\zeta;\zeta')], \quad \text{(S2)}$$

$$I_B = \frac{I_0}{8}[e^{i\zeta}(1+e^{i\varphi}) - e^{-i\zeta'}(1-e^{i\varphi})][e^{-i\zeta}(1+e^{-i\varphi}) - e^{i\zeta'}(1-e^{-i\varphi})]$$

$$= \frac{I_0}{8}[(1+e^{i\varphi})(1+e^{-i\varphi}) + (1-e^{i\varphi})(1-e^{-i\varphi}) - e^{i(\zeta;\zeta')}(1+e^{i\varphi})(1-e^{-i\varphi}) - e^{-i(\zeta;\zeta')}(1-e^{i\varphi})(1+e^{-i\varphi})]$$

$$= \frac{I_0}{8}[2 + 2cos\varphi + 2 - 2cos\varphi - e^{i(\zeta;\zeta')}(2isin\varphi) + e^{-i(\zeta;\zeta')}(2isin\varphi)]$$

$$= \frac{I_0}{2}[1 + sin(\varphi)sin(\zeta;\zeta')], \quad \text{(S3)}$$

where $(\zeta;\zeta')$ stands for (ζ) or (ζ') at a random basis.

2. Analysis of entangled inputs between E_α and E_β in Fig. 1(a).

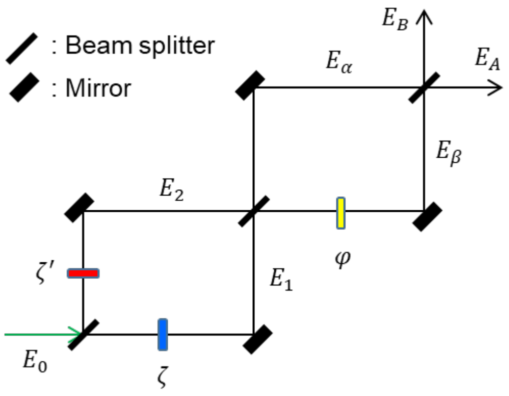

Fig. S1. Schematic of macroscopic entangled field generation (see Fig. 1(a)).

In Fig. S1, E_α and E_β are represented by matrix representations as follows:

$$\begin{bmatrix} E_\alpha \\ E_\beta \end{bmatrix} = \frac{E_0}{2}\begin{bmatrix} 1 & 0 \\ 0 & e^{i\varphi} \end{bmatrix}\begin{bmatrix} 1 & i \\ i & 1 \end{bmatrix}\begin{bmatrix} e^{i\zeta} & 0 \\ 0 & e^{i\zeta'} \end{bmatrix}\begin{bmatrix} 1 & i \\ i & 1 \end{bmatrix}\begin{bmatrix} 1 \\ 0 \end{bmatrix}$$

$$= \frac{E_0}{2}\begin{bmatrix} 1 & 0 \\ 0 & e^{i\varphi} \end{bmatrix}\begin{bmatrix} (e^{i\zeta} - e^{i\zeta'}) & i(e^{i\zeta} + e^{i\zeta'}) \\ i(e^{i\zeta} + e^{i\zeta'}) & -(e^{i\zeta} - e^{i\zeta'}) \end{bmatrix}\begin{bmatrix} 1 \\ 0 \end{bmatrix}$$

$$= \frac{E_0}{2}\begin{bmatrix} (e^{i\zeta} - e^{i\zeta'}) \\ ie^{i\varphi}(e^{i\zeta} + e^{i\zeta'}) \end{bmatrix}, \quad \text{(S4)}$$

where $e^{i\zeta}$ and $e^{-i\zeta}$ are alternative and 'off' party is replaced by 1, such that $E_\alpha = \frac{E_0}{2}(e^{i\zeta} - 1)$ or $E_\alpha = \frac{E_0}{2}(1 - e^{i\zeta'})$ according to the pulse sequence in Fig. 1(b). Likewise, that $E_\beta = \frac{iE_0}{2} e^{i\varphi}(e^{i\zeta} + 1)$ or $E_\beta = \frac{iE_0}{2} e^{i\varphi}(1 + e^{i\zeta'})$. Thus, $\langle I_\alpha \rangle = \langle I_\beta \rangle = I_0/2$ is satisfied for $\zeta = \pi/2$ and $\zeta' = -\zeta$. The final outputs E_A and E_B are dependent upon the inputs of E_α and E_β, satisfying a nonclassical features of equations (S2) and (S3). Here, our concern is about the input fields of E_α and E_β, whether they are entangled or not. It should be noted that $e^{i\zeta}$ and $e^{i\zeta'}$ in equation (S4) are not fixed but alternative, resulting in alternatively 'on' for both fields in each cycle of T (see Fig. 1(b)). Thus, both inputs E_α and E_β can be represented in a form of $|\psi\rangle = |E_\alpha\rangle|E_\beta\rangle$, and the following relation is obtained:

$$|\psi\rangle = \frac{iE_0}{\sqrt{2}}\left(|1\rangle_\alpha|0\rangle_\beta - ie^{i\varphi}|0\rangle_\alpha|1\rangle_\beta\right). \quad \text{(S5)}$$

Equation (S5) is a typical entanglement relation. Thus, the input fields E_α and E_β are entangled via random choice of the phase ζ in the first MZI.
